# A Recent Update on Advanced Molecular Diagnostic Techniques for COVID-19 Pandemic: An Overview

**DOI:** 10.3389/fimmu.2021.732756

**Published:** 2021-12-14

**Authors:** Akanksha Roberts, Raghuraj Singh Chouhan, Deepshikha Shahdeo, Narlawar Sagar Shrikrishna, Veerbhan Kesarwani, Milena Horvat, Sonu Gandhi

**Affiliations:** ^1^ Department of Biotechnology (DBT)-National Institute of Animal Biotechnology (NIAB), Hyderabad, India; ^2^ Department of Environmental Sciences, Jožef Stefan Institute, Ljubljana, Slovenia

**Keywords:** SARS-CoV-2, diagnostics, RT-PCR, LAMP, biosensors, point of care, CRISPR-Cas

## Abstract

Coronavirus disease 2019 (COVID-19), which started out as an outbreak of pneumonia, has now turned into a pandemic due to its rapid transmission. Besides developing a vaccine, rapid, accurate, and cost-effective diagnosis is essential for monitoring and combating the spread of severe acute respiratory syndrome coronavirus 2 (SARS-CoV-2) and its related variants on time with precision and accuracy. Currently, the gold standard for detection of SARS-CoV-2 is Reverse Transcription Polymerase Chain Reaction (RT-PCR), but it lacks accuracy, is time-consuming and cumbersome, and fails to detect multi-variant forms of the virus. Herein, we have summarized conventional diagnostic methods such as Chest-CT (Computed Tomography), RT-PCR, Loop Mediated Isothermal Amplification (LAMP), Reverse Transcription-LAMP (RT-LAMP), as well new modern diagnostics such as CRISPR–Cas-based assays, Surface Enhanced Raman Spectroscopy (SERS), Lateral Flow Assays (LFA), Graphene-Field Effect Transistor (GraFET), electrochemical sensors, immunosensors, antisense oligonucleotides (ASOs)-based assays, and microarrays for SARS-CoV-2 detection. This review will also provide an insight into an ongoing research and the possibility of developing more economical tools to tackle the COVID-19 pandemic.

**Graphical Abstract:**
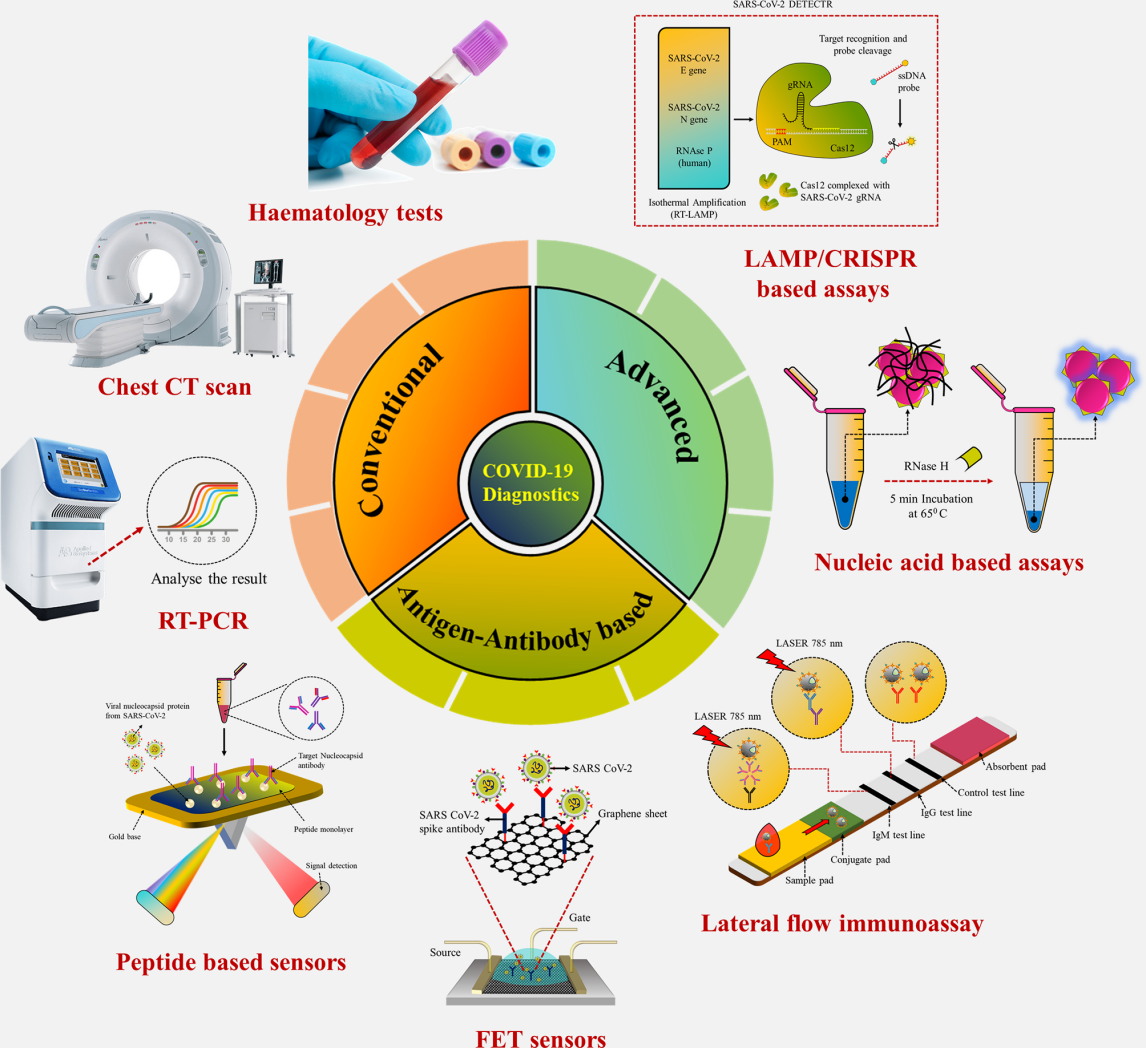
Conventional laboratory-based techniques for point-of-care detection of SARS-CoV-2.

## Introduction

Coronavirus was discovered in Wuhan, China due to an outbreak of pneumonia in December 2019 (1–5) and the World Health Organization (WHO) gave it the nomenclature 2019 novel coronavirus (2019‐nCoV). Based on a study on coronavirus, the International Committee on Taxonomy of Viruses, on comparing the phylogeny and taxonomy, declared this virus similar to severe acute respiratory syndrome coronavirus (SARS‐CoV) and named it SARS‐CoV‐2 (6). SARS-CoV was first reported as early as 2002 in Guangdong, China (1), while Saudi Arabia reported cases of MERS-CoV in June 2012. As of June 2021, a total of 176 million COVID-19 confirmed cases have been reported around the world. Transmission of SARS-CoV was primarily through direct contact with an infected person and had overall a low transmittable rate. Therefore, in June 2003, the outbreak was primarily contained within households, with only a few cases of infection and mortality being recorded. However, in stark contrast, SARS-CoV-2, which surfaced in December 2019, has turned into a pandemic.

**Figure 1:**
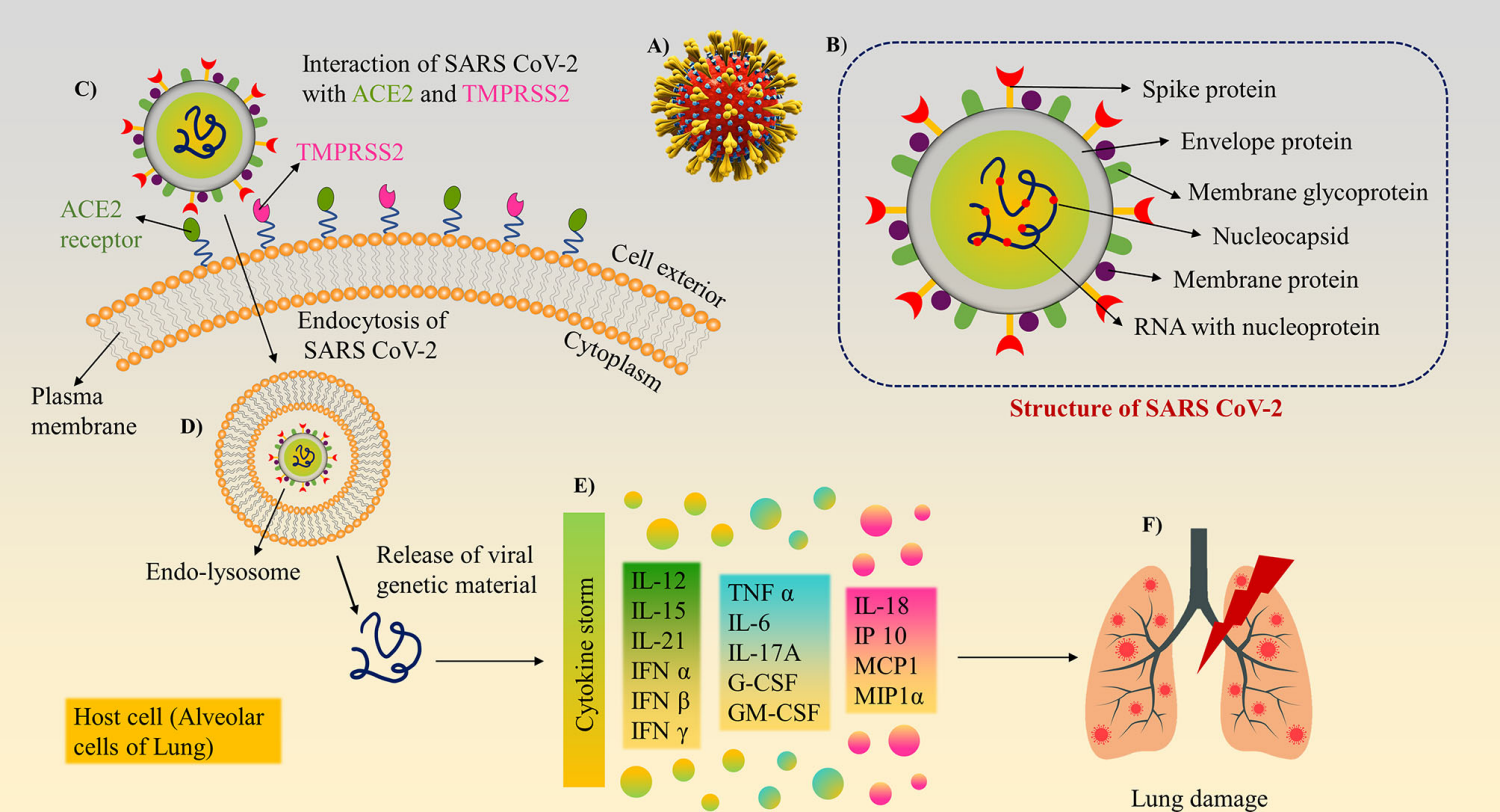
Structure and mechanism of action/pathogenesis of SARS-CoV-2: **(A)** 3D model of SARS-CoV-2 with surface spike glycoprotein. **(B)** Different structural and non-structural proteins of SARS-CoV-2, as a target biomarker for the development of diagnostics. **(C)** SARS-CoV-2 binds to ACE2 and Transmembrane protease serine 2 (TMPRSS2) receptors present on lung cells using its surface spike glycoprotein and internalised. **(D)** The virion further releases the viral genetic material into the cytoplasm. **(E)** Cytokine storm induction inside the cells. **(F)** Inflammatory response cause tissue/organ damage in the lungs.

SARS CoV-2 is a beta-coronavirus with a crown-like appearance due to the presence of spike S1 glycoprotein on its surface (7). Members of this family cause hepatic, enteric, respiratory, and neurological diseases in humans as well as different animal species, including bats, cattle, cats, and camels (8). The complete description of human Coronaviridae family and Orthocoronavirinae subfamily have been listed in **Table 1**. Coronavirus is a positive, single-stranded ribonucleic acid (RNA) (+ssRNA) virus (30 kb in length) that contains the following major structural proteins, i.e., spike (S), nucleocapsid (N), envelope (E), and membrane (M) protein as shown in **Figures 1A, B** (15).

**Table 1:** Different genus, subgenus, species, and genome size of human Coronaviridae family and Orthocoronavirinae subfamily.

Genus	Subgenus	Species (Abbreviations)	Genome Size	Reference
**Alphacoronavirus**	Duvinaco virus	Human coronavirus (HCoV-229E)	27,317	(9)
	Setraco virus	Human coronavirus (HCoV-NL63)	27,553	(10)
**Betacorona virus**	Embeco virus	Beta coronavirus (HCoV-OC43)	30,738	(11)
		Human coronavirus (HCoV-HKU1)	29,926	(12)
	Merbeco virus	Middle East respiratory syndrome-related coronavirus (MERSr-CoV)	30,119	(13)
	Sarbeco virus	Severe acute respiratory syndrome-related coronavirus (SARSr-CoV)	29,751	(14)

Among the structural proteins, spike protein comprises two subunits, S1 and S2, to guide viral attachment, fusion, and entry. S1 corresponds to the large receptor binding domain (RBD), while S2 is helpful in the formation of the stalk of the spike protein. RBD present on S1 binds to the Angiotensin-converting enzyme 2 (ACE-2) receptor enabling entry of the virus and Transmembrane protease serine 2 (TMPRSS2) receptor enabling S protein priming (16), which in turn is essential for viral transmission and pathogenesis. Other than the spike protein, the most abundant M protein has three transmembrane domains, which help shape the virion. E proteins are primarily found in small quantities and facilitate the assembly and release of the virus. N protein is composed of two domains capable of binding to the RNA of the virus. Hemagglutinin-esterase (HE) is the fifth structural protein present in β-coronaviruses, which has been recently discovered. It acts as a hemagglutinin and binds to sialic acid, thus enhancing the S-protein-mediated cellular entry of the virus. SARS-CoV-2 predominantly affects the airways and the epithelial cells of the lungs (17) and causes massive apoptosis of the epithelial and endothelial cells (18). Viral infection and replication in the body causes aggressive inflammation, and acute lung injury, along with the secretion of a cytokine storm of interleukins (IL-1β, IL-10, and IL-4), interferon (IFN-γ), IFN-γ produced protein (IP-10), and monocyte chemoattractant protein (MCP-1), as shown in **Figures 1C**–**F**.

Currently, there are multiple vaccines that are under various stages of development and trials, with mass vaccination drives underway in most countries. However, the transmission and evolution rate of the virus is much faster than the rate of vaccinating the entire world’s population, and hence, quick diagnostics is essential to manage the ongoing pandemic. Currently, there are mainly three types of diagnostic techniques available, Chest Computed Tomography (CT), RT-PCR (gold standard), and lateral flow-based chromatographic strip (19). CT scan and RT-PCR are limited to larger hospitals, are significantly time-consuming, and call for the requirement of skilled professionals (20). Moreover, CT scan can only detect the symptoms of the disease, but it cannot identify the specific virus strain present on the basis of these symptoms unlike in the way artificial intelligence (AI) can (21). However, antibody-based detection is currently unreliable for detecting early and asymptomatic cases (22). Hence, antibody-based tests, along with RT-PCR are being used in a joint effort to gain quick, accurate, specific, and highly sensitive COVID-19 diagnostic techniques. Herein, sensitivity is the ability of an assay to identify individuals with the disease (true-positive rate) on the basis of the lowest quantity of the given analyte that the assay can detect, whereas specificity is the ability of the assay to correctly identify those without the disease (true-negative rate) by reducing cross-reactive results. Accuracy is the umbrella term for both sensitivity and specificity that relates to the ability of the assay to correctly discriminate between the diseased and healthy individual.

To overcome certain limitations posed by conventional techniques, the development of different types of biosensors/biochips have begun to replace the conventional methods (23). Various bio-recognition elements such as aptamers, nucleic acids, peptides, antigens (Ags), and antibodies (Abs), can be immobilized on the sensors for highly specific detection of various biomarkers (24). Deployment of nanomaterial, such as gold and silver nanoparticles, graphene, quantum dots, carbon nanotubes, and molecularly imprinted polymers can further enhance the sensitivity of the diagnostic assay (25–30). This review article focuses on giving a brief overview of the current detection methods and provides a base for further developing highly efficient portable sensors for SARS-CoV-2 detection.

## Conventional Diagnostic Techniques

Currently, RT-PCR, CT scan, and hematology assays are the basic diagnostic tests for SARS-CoV-2 and are deemed as the gold standards. However, multiple serological assays are under development for quick diagnosis of COVID-19 but have the disadvantage of sensitivity as compared to the conventional techniques currently being used. However, it was observed that, when serological tests were combined with molecular tests, both sensitivity and specificity increased by several folds.

### Chest CT Scan

CT scan involves taking serial x-ray images from various angles and combining them, following which a computer program creates a cross-section image of the lungs. It is used to detect pneumonia caused by COVID-19 infection by demonstrating typical radiographic features including consolidated multifocal patchy, ground-glass opacity, and/or interstitial differences distributed peripherally (31). Patients displaying clinical symptoms but negative RT-PCR results may be diagnosed by typical CT features. A positive correlation was found between C-reactive protein, lactate dehydrogenase, and sedimentation rate of erythrocytes with the severity of pneumonia as seen *via* CT (32). Deep learning (DL) extracts CT images and divides the lesion area into segments, splitting COVID-19 into 6 stages: healthy, ultra-early, early, rapid progression, consolidation, and dissipation (33). CT is preferred over other techniques due to its high resolution, efficacy, sensitivity, and its reliability in evaluating the severity of respiratory infection. Bilateral thickening and irregular pleural lines are some of the patterns observed in the case of COVID-19. A more advanced version being used to detect low levels of lung infection in COVID-19 patients is high-resolution computed tomography (HRCT). In contrast to helical CT, HRCT uses narrow beam collimation to capture thin slice images of the lung parenchyma and provides higher definition images of lung alveoli, airways, interstitium, and pulmonary vasculature. However, it is a costly technique, and available only in central hospitals. This technique also has other limitations—it fails to distinguish between different viruses, and is of no use in asymptomatic cases.

### RT-PCR

**Figure 2:**
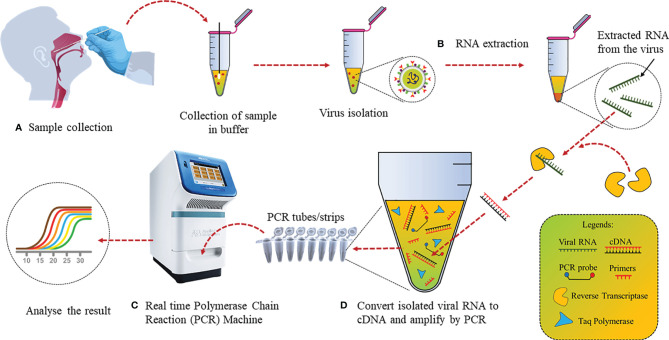
Detection of SARS-CoV-2 by conventional RT-PCR: **(A)** Nasopharyngeal swab samples collection in media and virus isolation. **(B)** RNA extraction from isolated virus using an RNA extraction kit. **(C)** Conversion of RNA to cDNA using reverse transcriptase. **(D)** cDNA PCR amplification with specific primers and fluorescent hydrolysis probes followed by analysis.

The detection of trace amounts of gene transcripts *via* RT-PCR has made it the ideal choice for various contagious disease diagnostics (34, 35). Several laboratories have been developing diagnostic assays, but RT-PCR remains the most widely used detection technique for SARS-CoV-2 as it directly measures parts of the viral genome or viral transcripts instead of secondary biomarkers. The genes such as *N*, *E*, and RNA-dependent RNA polymerase (*RdRp*) of SARS-CoV-2 are used for targeted detection *via* RT-PCR (36). RT-PCR fundamentally involves 2 steps: (a) reverse transcription of genome RNA or individual viral transcripts to form complementary DNA (cDNA) using an enzyme; (b) cDNA amplification by PCR using specific primers and labeled with fluorescent hydrolysis probes (**Figure 2**).

cDNA templates are produced in Step 1 and the yield is increased by repeating the thermal cycles. This template is then used for Step 2. Gene-specific primers amplify the selected region of the cDNA template, whereas the probes give fluorescent signals every time specific gene regions are amplified, resulting in quantifiable data (37). On January 11, 2020, as soon as the entire genome sequence of SARS-CoV2 was uploaded, Malaysian Institute for Medical Research (IMR) developed RT-PCR primers and probes for the same, while several other countries developed RT-PCR kits. A list of primers used by different research groups have been listed in **Table 2**.

**Table 2:** Primers available for different target genes of SARS-CoV-2 for RT-PCR.

Detection Method	Target Gene	Forward Primer	Reverse Primer	Reference
**TaqMan RT-PCR**	Nucleocapsid	TAATCAGACAAGGAACTGATTA	CGAAGGTGTGACTTCCATG	(38)
**TaqMan RT-PCR**	RdRp/Helicase	CGCATACAGTCTTRCAGGCT	GTGTGATGTTGAWATGACATGGTC	(39)
**TaqMan RT-PCR**	Spike	CCTACTAAATTAAATGATCTCTGCTTTACT	CAAGCTATAACGCAGCCTGTA	(39)
**TaqMan RT-PCR**	Nucleocapsid	GCGTTCTTCGGAATGTCG	TTGGATCTTTGTCATCCAATTTG	(39)
**TaqMan RT-PCR**	RdRp gene	GTGARATGGTCATGTGTGGCGG	CARATGTTAAASACACTATTAGCATA	(40)
**TaqMan RT-PCR**	Envelope	ACAGGTACGTTAATAGTTAATAGCGT	ATATTGCAGCAGTACGCACACA	(40)
**TaqMan RT-PCR**	Nucleocapsid	CACATTGGCACCCGCAATC	GAGGAACGAGAAGAGGCTTG	(40)
**TaqMan RT-PCR**	ORF1b	TGGGGYTTTACRGGTAACCT	AACRCGCTTAACAAAGCACTC	(40)
**TaqMan RT-PCR**	NSP2	ATGCATTTGCATCAGAGGCT	TTGTTATAGCGGCCTTCTGT	(41)

Recently, due to the increasing need for bulk sample testing, pooled sample testing for RT-PCR has also been approved. In 1943, this method of mixing several samples together in a “batch” or pooled sample was introduced by Dorfman and proved helpful in correctly identifying all infected individuals using fewer resources. RT-PCR being a robust technology with high sensitivity and specificity has already been used to test sample pools for human immunodeficiency virus and hepatitis B and C and is also now being employed for SARS-CoV-2. This is an effective method for large-scale screening of the population without wastage of resources and time. Even though RT-PCR is the most widely used SARS-CoV-2 diagnostic technique so far, it is time-consuming, and hence, the number of tests that can be carried out in a 24-h time period is low. This is a limiting factor that prevents reliable data of viral transmission to be collected in the entire population so as to gain an understanding of the world scenario. Due to low viral titer in the early days of the infection, RT-PCR may not be sensitive enough to detect the small amounts of RNA sample, leading to false-negative results. Therefore, multiple tests of different body fluids may be required for a confirmatory test, and a combination of RT-PCR with serological tests for confirmation.

### Hematology Tests

Blood tests have played a vital role in disease diagnosis over the years, and hematology tests, which include the study of blood samples, have also been introduced for the diagnosis of SARS-CoV-2. In case of SARS-CoV-2, blood profiling of COVID-19-positive cases showed lymphopenia and increased lactate dehydrogenase (LDH), and these patients were critical in the intensive care unit (ICU). Patients admitted to the ICU had a higher nadir absolute lymphocyte count (ALC), nadir absolute monocyte count (AMC), nadir hemoglobin (Hb), higher peak absolute neutrophil count (ANC), and high LDH levels as compared to less critically ill patients who did not require ICU admission (42). Other hemagglutination tests (HAT) have also been performed to detect SARS-CoV-2 antibodies to study seroconversion in the population. These simple, inexpensive and rapid tests rely on the hemagglutination activity of the virus and the ability of specific antibodies to inhibit the virus from agglutinating the erythrocytes (43). Townsend and group have reported a HAT that shows 90% sensitivity and 99% specificity in the detection of Abs after RT-PCR. This is comparable to commercially available kits for receptor-binding domain (RBD) Ab detection, and can be applied as point-of-care testing (POCT) (44). Kruse et al. tested the possibility of using the red blood cell agglutination assay for SARS-CoV-2 Ab detection. They created a fusion protein consisting of RBD from spike protein and scFv targeting H antigen on red blood cells. The agglutination test was performed by mixing the fusion protein with red blood cells and the serum of a patient recovered from COVID-19 (specimen collected at least 28 days post symptoms and a negative PCR test upon discharge). Optimal agglutination was achieved at the highest fusion protein concentration, i.e., 100% within 5 min of incubation (45).

## Novel Approaches for SARS-CoV-2 Diagnostics

### Nucleic Acid Based Assays for SARS-CoV-2

RT-PCR is a cumbersome and time-consuming process, requiring both expensive equipment and trained personnel. Moreover, it does not have the screening capacity to keep pace with the rapidly growing pandemic. Hence, an alternative technique is the Loop-Mediated Isothermal Amplification (LAMP) method, which is highly similar to RT-PCR in terms of sensitivity and specificity, but with one exception, the nucleic acid amplification occurs at a single temperature (isothermal amplification), and hence, there is no need for a thermocycler, which, in turn, makes the process faster (46). In the LAMP process, amplification of the gene occurs *via* two types of loop region elongation reactions (i.e., self-elongation of template strand from the stem loop at 3’ terminals followed by binding and elongation of new primers to the loop region), which keep on repeating. Loopamp kit, based on the loop-mediated isothermal amplification (LAMP), was compared with RT-PCR for SARS-CoV-2 detection. A total of 76 nasopharyngeal samples showed 100% sensitivity, 97.6% specificity, and limit of detection (LOD) up to 1.0 × 10^1^ copies/μl. Using four primers to detect six various regions in the RNA increased the sensitivity of this test (47). Reverse Transcription Loop-mediated isothermal amplification (RT-LAMP) method for SARS-CoV detection employed four primers to increase the assay sensitivity, and a fluorescent dye was used to determine the amplified product without any sample preparation (48). However, it needs a laboratory setup, and only one sample can be run at a time. RT-LAMP was done in a single 30-min step at 63°C and optical density (OD) was measured at 400 nm (with color changing to green from orange). The assay could identify *N*, *E*, and *Open Reading Frame-1ab* (*ORF1ab*) genes simultaneously with 92.3%, 98.5%, and 99% accuracy, respectively. All three genes were combined and amplified, and the results were correlated with RT-PCR test results on 208 clinical samples (20-fold dilutions) with LOD up to 1000 copies/ml. Another RT-LAMP assay could detect SARS-CoV-2 in 30 min (49). The GenBank MN908947 strain which showed high similarity to other SARS-CoV-2 strains but not with SARS-like bat coronavirus, was used to design six primers for RT-LAMP. To avoid tetraplex structure formation, which may interfere with RT-LAMP, the primers were made such that no four guanines came in a row. According to the gel electrophoresis data, 63°C for 30 min was the optimum condition for all six primers. The results matched with RT-PCR and LOD was approximately 1.02 fg. Various types of spiked samples, including urine, saliva, serum, nasopharyngeal, and oropharyngeal swabs, were used together with clinical urine, and plasma without any treatment. Urine samples showed more significant signals, and no cross-reactivity. When three colorimetric isothermal amplification methods [LAMP, cross-priming amplification (CPA), and polymerase spiral reaction (PSR)] were compared, LAMP showed the best results in naso- and oropharyngeal swabs with the highest sensitivity (43 copies) (50). LAMP or RT-LAMP based techniques are highly successful in SARS-CoV-2 detection. Since these techniques have certain practical limitations, these can be overcome using novel diagnostic methods such as CRISPR or antigen/antibody-based rapid detection kits.

The most widely applied diagnostic test for SARS-CoV-2, i.e., RT-PCR, is based on nucleic acid. Research is being carried out to develop rapid diagnostic sensor assays based on a similar principle of detecting viral nucleic acid. The fundamental basis of nucleic acid-based assays is the process of hybridization of a pair of nucleic acid, i.e., DNA/DNA or cDNA/RNA, based on their dependence as complementary sequences (51). They primarily make use of DNA, RNA, peptide nucleic acid (PNA), and aptamers (both DNA and RNA) as oligonucleotide probes. The basic principle of DNA, RNA, and PNA is based on sequence complementarity as per Chargaff’s rules of base pairing. However, in the case of aptamers, the principle is based more on receptor–ligand interactions. **Table 3** compares different nucleic acid-based point-of-care diagnostic kits for SARS-CoV-2, which follow different principles such as RT-PCR, LAMP, and RT-LAMP.

**Table 3:** Commercially available PoCT kits for SARS-CoV-2 detection with nucleic acid target analytes.

Detection Method	Target Analyte	Sensitivity (%)	Specificity (%)	Detection Time	Reference
**RCA with magnetic nanoparticles**	Synthetic complementary DNA (RdRp)	N/A	N/A	<2 h	(52)
**CRISPR-based LAMP with lateral flow assay**	RNA (E, N genes)	95	100	<1 h	(53)
**Real-time qRT-PCR**	RNA (RdRp, E, N genes)	100	100	>4 h	(40)
**Reverse transcription-LAMP**	RNA	N/A	N/A	<1 h	(49)
**LAMP with colorimetric readout**	RNA	N/A	N/A	<1 h	(54)
**Digital PCR**	RNA	N/A	N/A	<1 h	(55)
**Reverse transcription-LAMP**	RNA (ORF1ab, S genes)	100	100	<1 h	(56)
**Reverse transcription-LAMP**	RNA	N/A	N/A	<1 h	(57)

N/A, not available.

**Figure 3:**
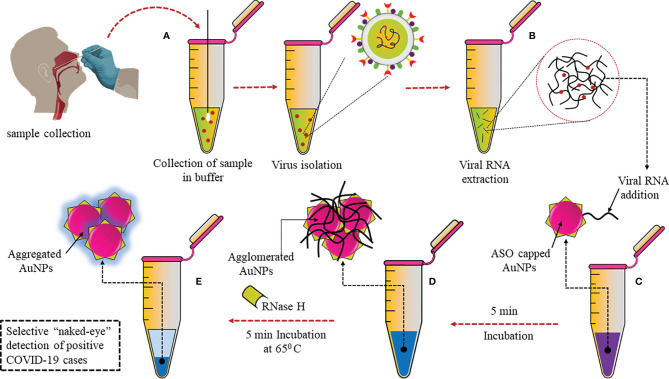
Selective naked eye SARS-CoV-2 detection: **(A)** Nasopharyngeal swab sample collected and virus isolated. **(B)** Viral RNA extraction. **(C)** Addition of AuNPs conjugated with thiolated ASOs specific for N gene. **(D)** Aggregation of ASO-AuNPs when target N-gene is present. **(E)** Addition of RNaseH to cleave RNA from RNA–DNA conjugate resulted in aggregation.

A plasmonic sensor with dual functions has been developed, combining localized surface plasmon resonance (LSPR) with plasmonic photothermal (PPT) effect. In this sensor, cDNA bioreceptors were functionalized onto two-dimensional gold nanoislands (AuNIs) to detect specific sequences of the SARS-CoV-2 genome through hybridization of nucleic acids. At its plasmonic resonance frequency, the AuNIs chip was illuminated, and thermoplasmonic heat was generated for better performance. This heat enhanced the temperature of *in situ* hybridization and ensured sensitive and specific differentiation between two gene sequences that may be similar. This dual-functional sensor had a very low LOD of 0.22 pM and was highly sensitive towards SARS-CoV-2, which allowed precise detection in a mixture of similar genes (58). Moitra et al. have developed a colorimetric assay that the naked eye can detect, as can be seen in **Figure 3**. Thiolated antisense oligonucleotides (ASOs) specific for N-gene are functionalized onto gold nanoparticles (AuNPs). The thiolated ASO-AuNPs aggregate when the target RNA sequence (N-gene) is present and a change in the surface plasmon resonance (SPR) can be detected within 10 min in the RNA extracted from clinical samples. Furthermore, RNA was cleaved from the RNA–DNA conjugate by adding RNaseH, which resulted in precipitation that could be visually detected due to the aggregation of AuNPs. This developed assay had negligible cross-reactivity with MERS-CoV and LOD at 0.18 ng/μl (59).

### Peptide-Based Assays for SARS-CoV-2

**Figure 4:**
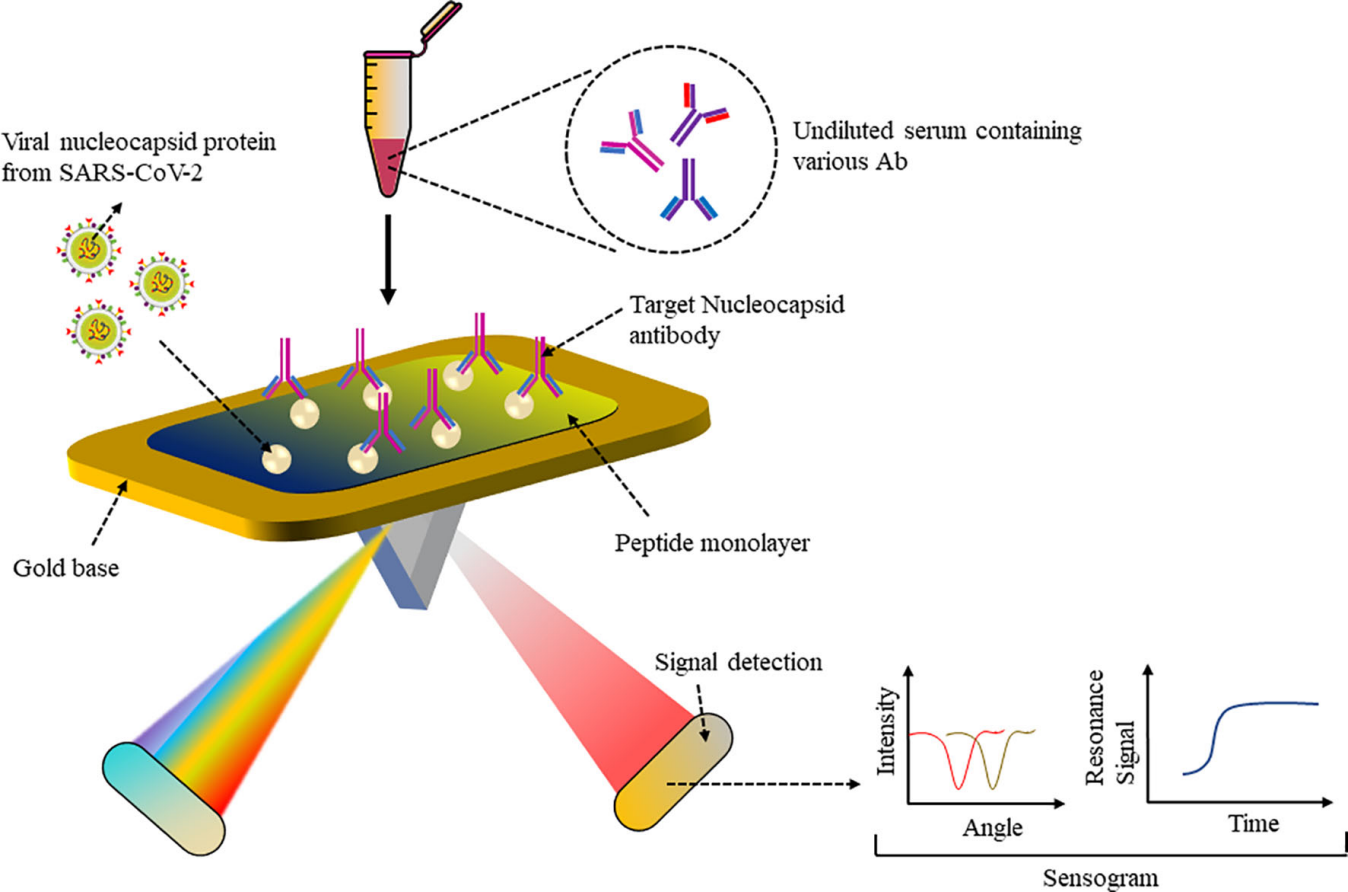
SPR-based sensor: Peptide monolayer functionalized with N protein coated onto gold base of SPR sensor. Addition of the clinical serum samples led to Ag–Ab interaction and change in SPR signal was recorded to detect SARS-CoV-2 Ab.

A biomimetic biosensor has an artificial bioreceptor component that can mimic natural bioreceptor activity and can be developed based on peptide bioreceptors (60). Numerous living organisms produce anti-microbial peptides (AMPs) to neutralize invading pathogens (61). For example, the AMP Cecropin-A from the *Hyalophora cecropia* moth, the first AMP to be isolated and characterized, shows inhibitory activity against herpes simplex virus 1 and 2 as well as human immunodeficiency virus 1. These short (mainly < 40 amino acids), amphipathic (contain both positively charged as well as hydrophobic residues), cationic proteins can have different secondary structures that can bind to receptors. Since they have a common building block, peptides with a specific sequence may be substituted for proteins in biological analysis. The advantage of using peptides is their smaller protein structure, making them more stable when coated on a biosensor and less prone to degradation rather than the entire large protein. Moreover, artificial peptides have various desirable properties such as chemical and structural versatility, high affinity to proteins or any analyte, standardized synthesis protocol and easy modification and hence make good bioreceptors for fabrication of biosensors. The surface plasmon resonance (SPR) sensor was able to detect nucleocapsid antibodies (Abs), which are specific for SARS-CoV-2. Abs expressed against SARS-CoV-2 N protein upon infection were detected in a number of patients immunized against SARS-CoV-2, which can be used to support vaccine research (62). As shown in **Figure 4**, a peptide monolayer functionalized with recombinant N Ag was coated onto the SPR sensor, to detect anti-SARS-CoV-2 Abs in nM concentration. The assay was conducted on undiluted human serum and a portable SPR machine was used to detect the assay results, which were obtained within 15 min. This strategy enables further research on label-free rapid point-of-care testing for Abs.

### CRISPR-Based Diagnostics for SARS-CoV-2

**Figure 5:**
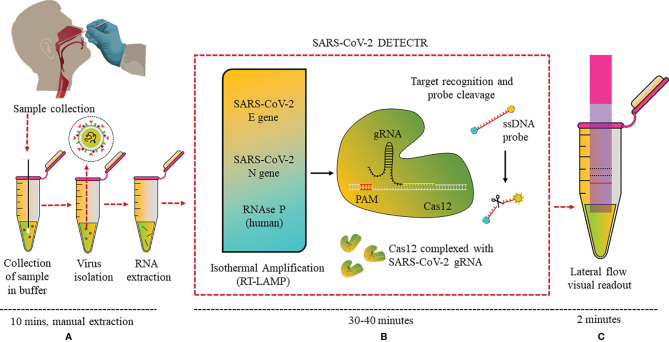
DETECTR LFA for SARS-CoV-2: **(A)** Nasopharyngeal swab viral RNA extracted from isolated virus to be used as the input sample or the sample matrix in the DETECTR assay. **(B)** RT-LAMP pre-amplification of E, N, and RNase P gene using ssDNA probes followed by Cas12-based detection. **(C)** Visual readout of the results on lateral flow strip by a fluorescence reader.

CRISPR (Clustered Regularly Interspaced Short Palindromic Repeats) technology has not only revolutionized genome editing, but also helped in the development of advanced diagnostic assays. CRISPR systems are an essential part of the adaptive immune system of microbes and has the ability to recognize foreign nucleic acids based on their sequence and remove them *via* endonuclease activity of the CRISPR-associated (Cas) enzyme, and this property has been made use of in the diagnostic field. CRISPR-Cas-based biosensors target a specific sequence in the genome, making use of CRISPR-Cas protein–RNA complex as the bioreceptor for specific recognition of the sequence (63). Combining RT-LAMP, CRISPR, and LFA in one assay, a detection kit was reported from the California Department of Public Health, University of California, Mammoth Biosciences Inc., and Abbott Viral Diagnostics and Discovery Inc. (53). This “SARS-CoV-2 DETECTR” assay extracted RNA from samples and, using RT-PCR (10 min), increased the copy numbers of RNAse P, N, and E genes (62°C for 20–30 min). CRISPR-Cas12 enzyme detected copies of the specific sequences of the genome and produced a fluorescence signal when the reporter dye was cleaved (37°C for 10 min) (**Figure 5**).

Eleven COVID-19 and 12 influenza nasopharyngeal swab samples were tested on this optimized assay. SARS-CoV-2 DETECTR proved to be 90% sensitive and 100% specific, and the results matched with that of the Centers for Disease Control and Prevention (CDC) RT-PCR standard assay. Moreover, this assay could specifically differentiate between SARS-CoV-2, SARS-CoV, and MERS-CoV with a single-nucleotide difference due to the specific primers and probe designed for N gene. The group is now focused on clinical validation of the kit for Emergency Use Authorisation (EUA) from the FDA. Similarly, Ding et al. have made an all-in-one dual CRISPR-Cas12a assay (AIOD-CRISPR) targeting the N protein gene and can detect even a few copies of the nucleic acids (DNA or RNA) (64). A PoC lateral flow diagnostic kit named FnCas9 Editor Linked Uniform Detection Assay (FELUDA) was developed using a Cas9 ortholog from *Francisella novicida*, for detection of single-nucleotide variants (SNV) in the nucleotide sequence of SARS-CoV-2 by the naked eye, which may be modified for diagnostics of various other diseases as well (65).

### Antibody Based Detection for SARS-CoV-2

**Figure 6:**
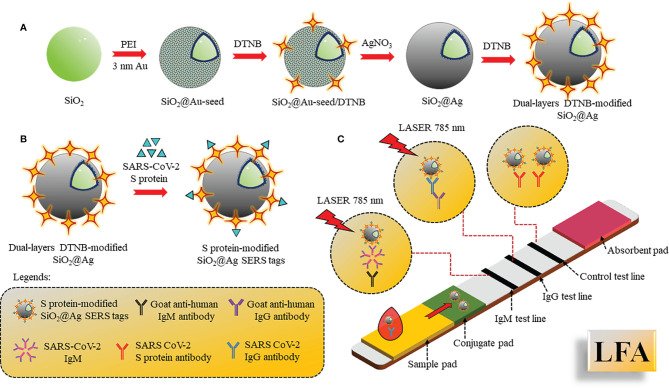
SERS-based LFA: **(A)** Preparation of double-layered DTNB-SiO_2_@AgNPs by PEI-mediated absorption method followed by addition of DNTB. **(B)** S protein conjugation with SiO_2_@Ag SERS tags. **(C)** LFA assembled with S protein-SiO_2_@Ag SERS tags drop casted onto the conjugate pad and 2 test lines coated with IgM, IgG, and control line coated with S protein Ab. The sample was applied on the sample pad and SERS signal intensities were recorded.

B lymphocytes of the adaptive immune system produce and secrete antibodies during the invasion of foreign pathogens. Since these antibodies are specifically produced against a specific antigen, the antibody–antigen reaction is highly specific and hence can be used as specific biomarkers for the diagnosis of diseases. Immune response against SARS-CoV-2 is triggered after a week that enhances production of immunoglobulins IgG/IgM in the blood to fight against the virus, and detection of these antibodies can help diagnose the infection. IgM is the first antibody to respond that helps eliminate the pathogens, while IgG provides the second, more robust, antibody-based immunity. As shown by Zhang et al., IgM and IgG can be detected after 5 days of infection by enzyme-linked immunosorbent assay (ELISA) (66). Pan and co-workers showed that the sensitivity of colloidal gold nanoparticle LFA for detection of IgG and IgM were 11.1%, 92.9%, and 96.8% within 7 days of infection, 8–14 days of infection, and after 14 days of infection, respectively (67). Corresponding to the above work, both IgG and IgM first appeared on the fourth day in confirmed cases, and positive detection of IgG and IgM were 3.6% and 11.1% respectively, in early stage. It was also observed that concentration of IgM stayed positive in approximately 75% intermediate and late-stage cases, whereas the IgG rate increased in the intermediate phase and rose to 96.8% in late-stage cases, leading to precise detection of IgG. Hence, detection of IgM and IgG in combination resulted in maximal testing efficacy, mainly during the intermediate stage. Furthermore, whole blood and plasma samples showed consistent results of 100% and 97.1% with excellent sensitivity in IgM and IgG assays (68, 69). A quick approach was investigated for the detection and targeting of viral IgG or IgM antibodies using colloidal gold-based immunochromatographic (ICG) assay and compared with RT-PCR (70). Label-free nanophotonic biosensor was developed, which can be integrated into small portable devices that could be given to different clinics (71). The LOD was approximately 0.2 pM, corresponding to 2 × 10^4^ copies of SARS-CoV-2. A multiplexed grafting-coupled fluorescent plasmonics (GC-FP) biosensor was designed for sensitive and rapid detection of Abs in dried blood spots and serum of humans (72). This GC-FP technique could measure Ag–Ab interactions at multiple targets simultaneously in one sample with 100% sensitivity and selectivity. Upon measuring IgG levels in the serum against three antigens of COVID-19, N protein, spike S1S2, and spike S1 yielded linear and quantitative response with dilution as low as 1:1600, and the results produced corresponded with a Luminex-based microsphere immunoassay and ELISA. The efficacy of dried blood samples had 100% selectivity and 86.7% sensitivity in COVID-19 diagnostics. This test was further conducted to detect multiple Ig isotypes with sensitive detection of IgG, IgM, and IgA. Another widely used method is lateral flow immunoassay (LFA). LFA is a simple cellulose-based device and has been considered as one of the most popular PoCT devices to detect the Ab present in the blood. It consists of a sample pad and conjugate pad, embedded onto a nitrocellulose membrane. Sample (blood, cells, and antibodies) is loaded onto the sample pad and allowed to move to the conjugate pad, where the antibody binds to pre-immobilized AuNPs-conjugated antigen (SARS-CoV-2 spike), and continues to move to the test and control lines due to capillary action. Molecules such as fluorophores, latex nanoparticles, and gold nanoparticles are used for the colorimetric visualization. Surface Enhanced Raman Spectroscopy (SERS)-based lateral flow immunoassay (LFA) using dual-layered 5,5’-dithiobis-(2-nitrobenzoic acid) DTNB-modified SiO_2_@Ag NPs, and SARS-CoV-2 S protein-modified SiO_2_@Ag SERS tags (**Figure 6**) were also reported to show ultra-sensitive and quick detection of IgM/IgG in clinical samples for diagnosis of COVID-19.

The dual-layered DTNB-modified SiO_2_@AgNPs was synthesized by the absorption of 3 nm Au seed NPs on SiO_2_ mediated by polyethyleneimine (PEI) and the addition of 5,5’-dithio-bis-(2-nitrobenzoic acid) (DNTB) to fabricate SiO_2_@Au-seed/DTNB NPs, from which SiO_2_@Ag core-shell NPs synthesis was carried out using seed growth technique by AgNO_3_ reduction using formaldehyde. The second layer of DTNB was modified in such a way onto the SiO_2_@AgNPs surface that it formed dual-layered DTNB-loaded nanotags. S protein was further conjugated with SiO_2_@Ag SERS tags. The LFA was assembled with S protein-SiO_2_@Ag SERS tags drop cast onto the conjugate pad and two test lines coated with anti-human IgM, IgG, and the control line coated with S protein Ab. Different concentrations of IgM/IgG were analyzed simultaneously by analyzing the intensity of SERS signal on the test/control lines (73).

### Antigen-Based Diagnostics for SARS-CoV-2

Targeting a specific antigen produced by a foreign pathogen either secreted in the circulatory system or on the surface of the pathogen is a highly specific method for detection of that pathogen. Since the results obtained are rapid, they are often used for a first laboratory investigation/screening in a suspected case. SARS-CoV-2 surface or secretory Ag can be detected in clinical samples within 2 days of infection, even when the viral load is low before the manifestation of symptoms, and hence it can help in early diagnosis, especially when compared to Ab-based detection, as antibodies develop later. For example, IgM can be detected in 5 days to 1 week and IgG after a week. A comparative **Table 4** has been listed for Ag and Ab-based diagnostic tests for SARS-CoV-2.

**Table 4:** Various antigen- and antibody-based diagnostic kits available for the detection of SARS-CoV-2.

Type of POCT Kit	Target Analyte	Sensitivity (%)	Specificity (%)	Detection Time	Reference
Graphene field effect transistor (Gra-FET)	Antibodies	N/A	N/A	N/A	(74)
LFIA	IgM and IgG	88.8	90.5	15 min	(22)
LFIA	IgM and IgG	97.7	N/A	N/A	(74)
Colloidal gold-based immune-chromatographic (ICG) strip	IgM or IgG	96.7	N/A	15 min	(67)
Chemi-luminescence immunoassay	IgM and IgG (recombinant nucleocapsid)	82.3	97.4	23 min	(75)
Immune-precipitation and parallel DNA sequencing	Antibody	90–97	~98	N/A	(76)
Colloidal Gold Immuno chromatographic assay (GICA)	Nucleoprotein	57.8	99.6	15 min	(77)
GICA	Nucleoprotein	30.3	100	15 min	(78)
Fluorescence immuno chromatographic assay (FICA)	SARS-CoV-2 antigen	93.8	100	<15 min	(79)
GICA	Nucleoprotein	50.5	100	N/A	(80)
Microfluidic FICA	SARS-CoV-2 antigen/IgG/IgM	N/A	N/A	<15min	(81)
Chemiluminescence Immunoassay (CLIA)	Nucleocapsid protein	55.3	99.7	30 min	(82)
FICA	Nucleocapsid protein	11.6	N/A	30 min	(83)
FICA	Nucleocapsid protein	67.8	100	10 min	(84)
GICA	IgG and IgM	86.88	99.40	5–10 min	(85)
GICA	IgG and IgM	95.10	91.3	15 min	(86)
CLIA	IgG and IgM	100	N/A	N/A	(87)
CLIA	IgG and IgM for nucleocapsid protein	81.54	96.62	N/A	(88)
ELISA	IgG and IgM	81.33	N/A	N/A	(89)
ELISA	IgG and IgM for nucleocapsid and spike protein	82.3	N/A	N/A	(90)
ELISA	IgA, IgM, and IgG	85.5	N/A	N/A	(91)
ELISA	IgG, IgA against spike protein	N/A	N/A	N/A	(92)

N/A, not available.

**Figure 7:**
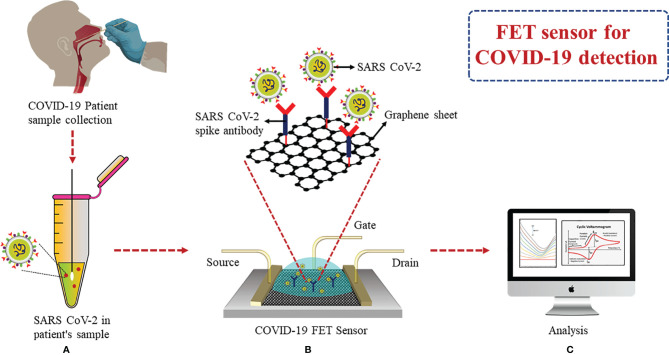
FET-based graphene sensor: **(A)** Collection of nasopharyngeal swab. **(B)** Immobilization of spike Ab *via* 1-pyrenebutyric acid N-hydroxysuccinimide ester on graphene FET sensor. **(C)** Analysis of binding events of Ag–Ab through electrical signals obtained for detection of spike Ag.

A FET biosensor using a graphene base was able to detect S protein and the complete virus in clinical samples. Spike Ab was immobilized onto the FET sensor *via* 1-pyrenebutyric acid N-hydroxysuccinimide ester for spike Ag detection (**Figure 7**). According to the results, LOD was found to be 1 fg/ml when prepared in phosphate buffer saline (PBS) and 100 fg/ml in clinical transport medium. It was demonstrated that SARS-CoV-2 showed an LOD of 1.6 pfu/ml when in culture and 2.42 copies/ml in clinical samples. It was possible to differentiate between SARS-CoV-2 spike protein from MERS-CoV in nasopharyngeal swabs (in the transport medium) using this FET sensor (93). A similar graphene-based FET sensor was developed by Zhang et al., which detected the target biomarker Spike protein S1 containing the RBD within 2 min in real time without any labeling and showed a highly sensitive LOD of 0.2 pM (94).

A nanostar and graphene-based electrochemical detection system was developed for SARS-CoV-2 in an aqueous solution, composed of Screen-Printed Carbon Electrode (SPCE). The SPCE working electrode was activated using graphene oxide (GO) and gold nanostars (AuNS), which can detect viruses in biological media (blood, saliva, and oropharyngeal/nasopharyngeal swab). The nanosensor demonstrated a superior LOD of 1.68 × 10^−22^ μg/ml in biological media, while blind testing of 100 clinical samples further proved the sensitivity and specificity (95). Mertens et al. developed a colloidal gold immunochromatographic assay where nucleoprotein MAbs-gold conjugate was coated on LFA. The Respi-strip was accurate by 82.6% and had overall sensitivity and specificity of 57.6% and 99.5%, respectively, and the results were obtained within 15 min on nasopharyngeal swab samples (77). A fluorescence immunochromatographic assay was developed for N protein detection in nasopharyngeal swab and urine samples in 10 min. The bioreceptor used on the test line was nucleocapsid MAb and carboxylate-modified polystyrene Europium (III) chelate microparticles conjugated with anti-N protein MAbs on the conjugate pad. In 73.6% of confirmed positive cases, the N Ag was detected in the urine on the same day (84). Another Fluorine Doped Tin Oxide (FTO)-based diagnostic electrode was fabricated to detect spike Ag in saliva samples. Spike Ab was immobilized on the electrode and FTO results were measured on a potentiostat. Under optimum conditions, the sensor showed a LOD of 0.63 fM and 120 fM in buffer and spiked saliva, respectively (96). Another low-cost electrochemical advanced diagnostic (LEAD) technology biosensor used inexpensive graphite leads modified with human angiotensin-converting enzyme 2 (ACE2) antigen as the receptor for on-site detection of SARS-CoV-2 Spike protein in clinical saliva and nasopharyngeal samples. The developed technology provided results within 6.5 min and showed an LOD of 229 fg/ml (97).

**Figure 8:**
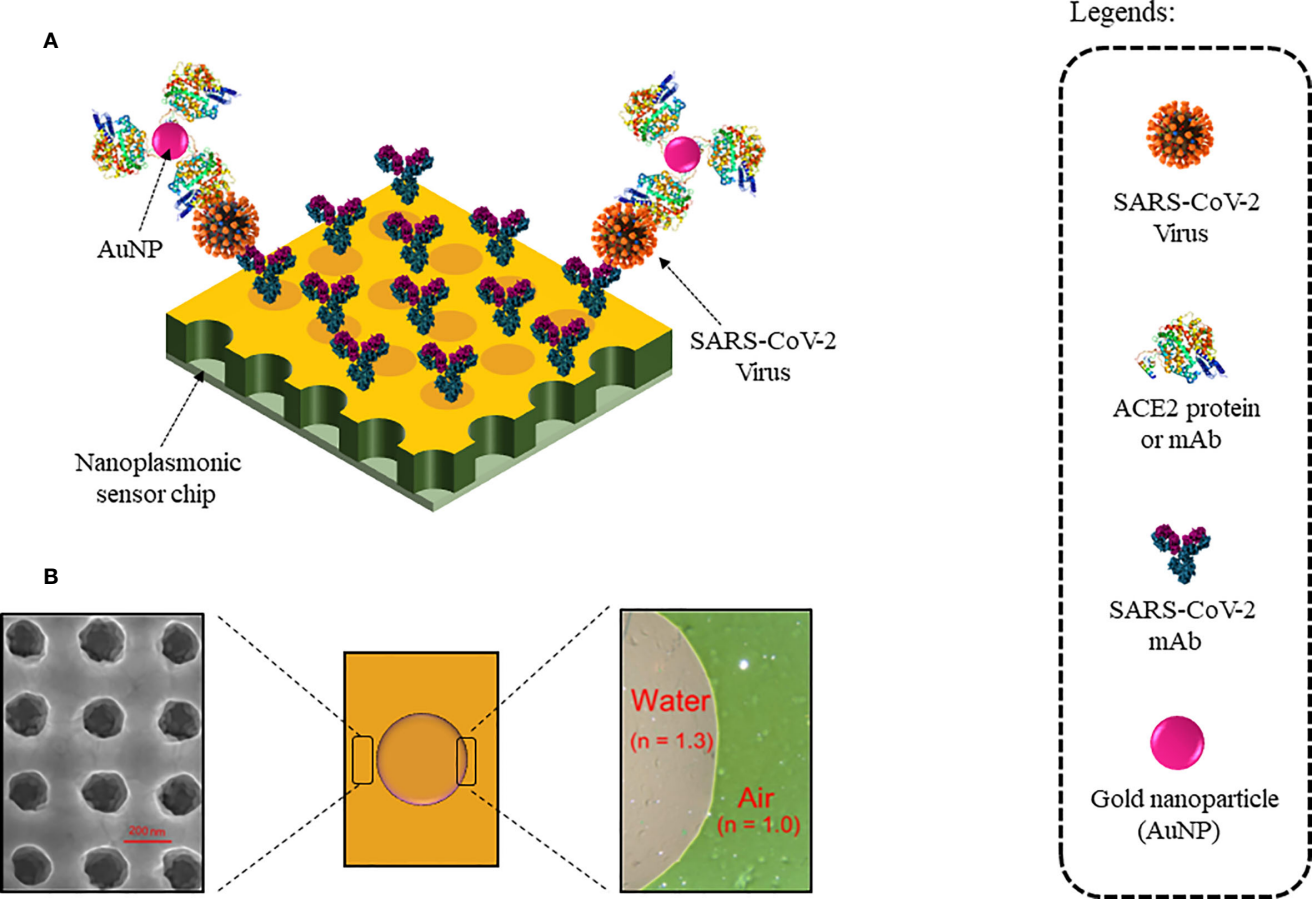
Nanoplasmonic Sensor for label-free detection of COVID-19: **(A)** Sensor chip coated with SARS-CoV-2 mAbs. Colorimetric detection with AuNPs conjugated with ACE2 protein or mAb. **(B)** Au nanocup array chip with a drop of water on top. (Left) Zoomed SEM image of the nanocup array. (Right) TEM image showed air and water interfaces exhibiting green and far red pink colors, respectively.

Nanoplasmonic sensors use nanoscale light below the *diffraction limit* (which typically is half the width of the wavelength of light being used to view the specimen) by converting free photons into localized charge-density oscillations (so-called surface plasmons) on noble-metal nanostructures, which serve as nanoscale analogs of radio antennas and are typically designed by using antenna theory concepts. One such nanoplasmonic resonance sensor coated with SARS-CoV-2 monoclonal antibodies (mAbs) was developed for a one-step direct optical rapid detection of SARS-CoV-2 virus particles, i.e., 15 min without any sample preparation with a LOD of 370 vp/ml as shown in **Figure 8**. Measurements could be taken on both a classic microplate reader and a handheld smartphone connected to the device, making it an excellent POCT (98).

As discussed above, conventional techniques, such as RT-PCR and a few commercial antibody detection assays, are the most prominently used diagnostic tests for the quick identification and immediate isolation of COVID-19 patients to prevent further transmission of COVID-19 infection. However, these assays have certain limitations, such as low specificity and sensitivity, elaborate sample preparation requirements that, in turn, demands skilled personnel, high response time, cumbersome protocols as most of them require a laboratory setup and equipment, and often expensive. The low viral load (less RNA) in asymptomatic patients and low levels of other potential biomarkers (e.g., Spike, Nucleocapsid, Envelope, and RdRp proteins) in body fluids for early screening of COVID-19 patients make point-of-care detection challenging. In comparison to the existing conventional techniques, recent detection methods such as antigen-based assays and peptide- and aptamer-based biosensors showed higher sensitivity, more specificity, and rapid detection time, as can be seen from the assays listed in **Tables 3** and **4**, and hence, can potentially overcome the limitations. These assays can be used to develop PoCT devices for large-scale sensitive screening of populations for SARS-CoV-2 especially when the titer is low such as in the case of asymptomatic patients or early stages of the disease.

## Conclusion

The knowledge gained from similar viral outbreaks of SARS-CoV and MERS-CoV in the past have led to the development of rapid diagnostics during the onset of SARS-CoV-2. Genome sequence identification of SARS-CoV-2 paved the way towards nucleic acid-based assays, which helped control the spread of COVID-19. The antibody and antigen-based methods provide further understanding, if compared with nucleic acid-based assays for COVID-19-infected patients. We have discussed the recent methods developed for SARS-CoV-2 diagnostics including its drawbacks. Currently, not much research exists in the development of peptide- and aptamer-based biosensors, which could lead to much higher sensitivity and specificity in SARS-CoV-2 detection. The diagnostic market has an immense potential to deal with the challenges associated with the existing methods for rapid and accurate detection at early stage of infection, which indeed is and must be the primary criteria. Keeping this view in our minds, we have emphasized on potential methods that are being used to control the transmission of SARS-CoV-2. However, these methods can further be employed for pandemic outbreaks in the future for swift and immediate control in hospitals, clinics, healthcare centers, and rural areas.

## Author Contributions

All authors listed have made a substantial, direct, and intellectual contribution to the work and approved it for publication.

## Funding

The authors would like to acknowledge the support provided by Science and Engineering Research Board-Intensification of Research in High Priority Area (SERB-IRHPA) (Grant Number-IPA/2020/000069). RC and MH gratefully acknowledge the Slovenian Research Agency (ARRS) through the funding of programme P1-0143. AR would like to acknowledge DST-INSPIRE fellowship IF180729 sponsored by DST, New Delhi.

## Conflict of Interest

The authors declare that the research was conducted in the absence of any commercial or financial relationships that could be construed as a potential conflict of interest.

## Publisher’s Note

All claims expressed in this article are solely those of the authors and do not necessarily represent those of their affiliated organizations, or those of the publisher, the editors and the reviewers. Any product that may be evaluated in this article, or claim that may be made by its manufacturer, is not guaranteed or endorsed by the publisher.
